# The Role of Soil Microorganisms in Microplastic Biodegradation: Mechanisms, Carbon Preferences, and Ecological Impacts

**DOI:** 10.1111/1758-2229.70270

**Published:** 2026-01-14

**Authors:** Mohammad Yaghoubi Khanghahi, Carmine Crecchio, Adriano Sofo, Rosangela Addesso

**Affiliations:** ^1^ Department of Agricultural, Forestry, Food and Environmental Sciences (DAFE) Università degli Studi della Basilicata Potenza Italy; ^2^ Department of Soil, Plant and Food Sciences University of Bari Aldo Moro Bari Italy

**Keywords:** biodegradation, bioremediation, carbon cycling, carbon use efficiency, microbial interactions, soil ecosystems

## Abstract

Microplastics (MPs) impact soil microorganisms by altering habitats and community structures. However, generalising these effects across polluted environments is challenging, particularly concerning soil carbon's role in biodegradation. This review aims to address crucial knowledge gaps regarding the relationship between soil carbon availability and microbial preferences for MP‐derived polymers. It highlights that, despite being carbon‐based, the unique structures of MPs prevent them from functioning like natural organic matter in the soil. This limitation affects both the degradation process and the ability of soil microorganisms to utilise MPs effectively as a carbon source. Notably, even polymers that are not directly assimilated after MP biodegradation can be transformed by other soil microorganisms into more readily exploitable forms through vital microbial interactions within the soil food web, which play a key role in carbon cycling. Moreover, this review emphasises attention on understanding how the microbial preferences for substrates derived from MPs are influenced by more readily available organic carbon in the soil. Evaluating carbon use efficiency among these communities reveals intricate responses of soil microorganisms to various carbon sources, including those from MPs. Overall, this review underscores the complex interplay between soil microorganisms, carbon sources, and MP pollution.

## Graphical abstract

This work explores the complex relationship between soil carbon availability and microplastic biodegradation, highlighting competition and symbiosis among microorganisms, the repurposing of microplastic‐derived polymers, and the impact of soil organic carbon on microbial substrate preferences and carbon use efficiency variability.

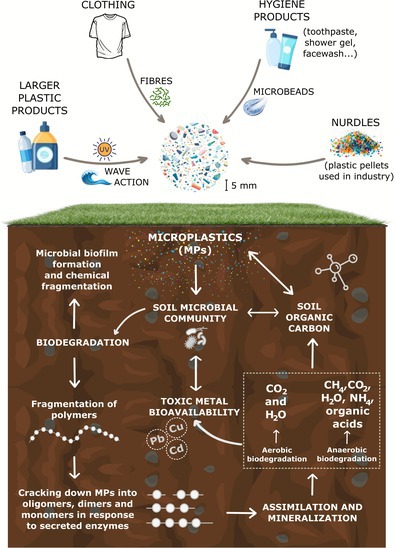

## 1 Introduction

Plastic pollution is a growing global concern that poses risks to both ecological security and public health, with unpredictable impacts (Addesso et al. 2022; Zhang, Li, et al. 2023; Balestra et al. 2024). Plastics can be decomposed into microplastics (MPs), which are tiny particles less than 5 mm in size, due to environmental factors such as weathering and erosion (Rillig and Lehmann 2020; Santini et al. 2023). The unique synthetic polymer structure of MPs results in notable environmental and ecological alterations in soil, influencing biophysical and biochemical properties that impact soil microorganisms and community dynamics (Sun, Duan, Cao, Li, et al. 2022; Rosati et al. 2024). Furthermore, MPs possess a high surface area and chemical stability, allowing them to adsorb various harmful substances, including persistent organic pollutants and heavy metals (Geyer et al. 2017; Santini, Maisto, et al. 2022). They can also facilitate the transport of pathogens and toxic by‐products (e.g., amines and phthalate esters), thereby exacerbating ecological and health risks (Dmytriw 2020; Zhang, Li, et al. 2023). Over time, the long‐term exposure of MPs to soil environments causes their surfaces to become increasingly rough, leading to a larger specific surface area and significantly enhanced adsorption capacity (Zhang, Tan, et al. 2022; Forster et al. 2023). This gradual deterioration suggests that the persistence and detrimental impacts of long‐term MP pollution in the environment will worsen, as their ability to attract and retain harmful substances grows stronger (Zhang, Tan, et al. 2022).

Furthermore, once ingested by organisms, MPs can move through the food chain, posing threats to biodiversity and the integrity of ecosystems (Wang et al. 2022). Their concentrations in the soil can vary widely, ranging from a few hundred to several thousand MP particles per kilogram, in which the common global MP concentrations in the soil can reach up to 13,000 particles kg^−1^ of dry soil (≈4.5 mg kg^−1^ of dry soil) (Büks and Kaupenjohann 2020). Specific values depend on factors such as land use, the origin of MPs, and local industrial or agricultural practices, such as the use of sewage sludge and wastewater, plastic mulching, and littering (Büks and Kaupenjohann 2020; Trojan et al. 2024). Table 1 summarises the effects of various types of MPs on soil physicochemical and biological properties as well as plant growth, highlighting the complex interactions between MPs and soil ecosystems.

**TABLE 1:** Effects of various MPs on soil physicochemical and biological properties, as well as plant growth across different soil types and conditions.

Soil structure/type	MP type	MP size	MP concentration	Duration of experiment (day)	Effects	References
Lime concretion black and silty loam	Polyurethane (PU)	Large (3.1 ± 0.8 mm) and small (56.3 ± 27.8 μm)	0.1% and 1.0% (w/w)	14 and 18	The physiological and morphological traits of maize plants grown in polluted soil were affected, with plant height, biomass, and pigment content increasing with exposure to larger PU particles and decreasing with exposure to smaller PU particles.	Zhang et al. (2024)
—	Polyethylene (PE) and polypropylene (PP)	PE: 6.5 ± 1.9 μm PP: 7.8 ± 1.6 μm	1%, (w/w)	35	MPs significantly impacted *Brassica juncea* , leading to early senescence and flowering. Furthermore, oxidative stress in the leaves was evidenced.	Wang et al. (2024)
Coastal saline‐alkali soil	PE and PP	—	0.1%, 1% and 5% (w/w)	120	A high concentration of PE enriched the composition of bacteria and fungi, with the effects of PE being more pronounced than those of PP. Additionally, fungi demonstrated a greater sensitivity to MPs compared to bacteria.	Yuan et al. (2023)
Sandy and loamy	PE	150 μm, 550 μm, and 950 μm	0.5%, 1%, and 2%	—	The addition of 2% MPs measuring 950 μm reduced the porosity of sandy soil, while the incorporation of 2% MPs measuring 150 μm led to an increase in the porosity of loamy soil.	Wang et al. (2023)
Mollisol soil	PE and biodegradable MPs	—	0.1%, 1%, and 5%	90	The processes of nitrogen fixation, nitrogen degradation, and assimilatory nitrate reduction in soil were stimulated.	Hu et al. (2023)
Sandy loam	Low‐density polyethylene (LDPE), polyester, PP, polyethylene terephthalate (PET), PU, and PS	< 5 mm	0.1%, 0.2%, 0.3%, and 0.4%	42	Foams and fragments improved soil aeration and microporosity. However, all shapes led to a reduction in soil aggregation, while films contributed to a decrease in soil bulk density.	Lozano, Lehnert, et al. (2021)
Sandy loam	Polyester	Length: 1.28 ± 0.03 mm, diameter: 0.030 ± 0.0008 mm	0.4%	60	Soil pH rose by approximately 4%, while soil aggregation improved by around 18%, and nutrient retention increased by as much as 70%.	Lozano, Aguilar‐Trigueros, et al. (2021)
Coastal saline‐alkali	PE and PP	—	0.1%, 1% and 5% (w/w)	120	MPs boosted enzyme activities and improved the nutrient content in the soil. High doses of PE inhibited the nitrogen fixation potential of soil bacteria, whereas high doses of PP enhanced it.	Yuan et al. (2023)
	PP and rubber crumb (RC)	100 μm	1%	10	MPs modified nitrogen cycling in the soil by decreasing available nitrogen, which includes both NH_4_^+^ and NO_3_^−^.	Liu et al. (2023)
Purple soil	PE	300 and 600 μm	5%	28	300 μm PE reduced dissolved organic matter and the activity of β‐1,4‐*N*‐acetylglucosaminidase, while also increasing cation exchange capacity.	Ma et al. (2023)
Typic Melanoxerand (Andisol)	HDPE	2–5 mm	0.2 g kg^−1^	180	A reduction was observed in the height, biomass, stem diameter, and root surface area of strawberry plants.	Pinto‐Poblete et al. (2023)
Sandy loam	PE, PS, and PP	150 μm	1%, w/w and 0.01%–20%, w/w of PP	60	MPs produced unique effects on soil bacterial communities in soil due to their varying polymer structures or chemical compositions. The bacterial community was notably influenced by PE and PP, in contrast to PS.	Sun, Duan, Cao, et al. (2022)
Andisol (medial, thermic Humic Haploxerands) silty	PS, PP, PET, LDPE	250 μm	2.5%, 5%, and 7% w/w	2	MPs were detected throughout all segments of the earthworms following ingestion, accompanied by a decrease in mucus production in their membranes.	Baeza et al. (2020)
Forest soil	PET and LDPE	56.3 ± 12.8 μm (PET) 25.3 ± 8.4 μm (LDPE)	0.2% and 0.4% (w/w) (PET) 3% (LDPE)	42	The introduction of PET at concentrations of 0.2% and 0.4%, as well as LDPE at 3%, led to a decline in both the diversity and abundance of the soil bacterial community.	Ng et al. (2021)

Recent studies have shown that MPs in the soil can affect soil microorganisms by disrupting their habitats. This disruption results in changes to taxonomic structures and influences potential interactions within microbial communities due to the toxicity of MPs (Rong et al. 2021; Sun, Duan, Cao, Li, et al. 2022). Furthermore, the community composition of bacteria and fungi in various soil aggregate fractions has undergone significant changes in response to long‐term exposure to MPs (Hou et al. 2021). Furthermore, there is a considerable body of literature focused on the biodegradation of MPs by soil microorganisms under both in vitro and in vivo conditions, making it one of the most promising areas of research in soil bioremediation (Alaidaroos 2023; Jain et al. 2023). These studies have introduced some soil bacteria and fungi species that have developed the ability to degrade certain types of plastics, leading to the formation of smaller organic compounds that can be assimilated into the microbial food web (Zhang et al. 2021; Li, Yang, et al. 2023).

Moreover, understanding the dynamics of microbial interactions and preferences in MP‐affected soils is crucial for effective bioremediation strategies. Given that MPs can alter nutrient availability and influence microbial community composition (Wang et al. 2025), the investigation of co‐metabolic processes becomes paramount. These processes, wherein microorganisms utilize primary substrates for growth while simultaneously transforming non‐essential compounds (Zhao et al. 2024), may enhance the biodegradation of MPs in contaminated environments. For instance, when organic carbon substrates are abundant, they could stimulate microbial activity and enzyme production (Zhao et al. 2016), leading to increased degradation rates of MPs. This relationship emphasizes the crucial need to explore how varying carbon sources, including both SOC and MP‐derived carbon, affect microbial growth and activity. Realising the importance of these interactions could significantly improve approaches to managing plastic pollution, ensuring that remediation efforts are tailored to optimise microbial function while sustaining soil health. In this light, a holistic understanding of the ecological impacts of MPs on soil microbial communities will shape future research directions and contribute to the sustainable management of soil ecosystems amidst growing plastic contamination.

However, the generalizability of much‐published research on the biodegradation potential of microorganisms in polluted environments across different MPs is problematic, as the role of soil carbon status and its interaction with microbial communities in contaminated soils are not well considered. What is clear from previous studies is the significant benefit of the substantial surface area of MPs in providing suitable ecological niches for the growth and sedimentation of those microorganisms using MPs as carbon sources (Li, Yang, et al. 2023). However, there is still much uncertainty about the mechanisms employed by soil microorganisms, their preference for carbon sources, and the potential ecological impacts of these interactions. Therefore, the key questions that have arisen here are as follows:

- If the availability of soil organic carbon (SOC) is high, do these MP‐degrading microorganisms still prefer to use MPs polymers as a carbon source for energy supply?

‐ If so, can shifts in MP degradation lead to modification in other factors in the soil, such as potentially toxic elements (PTEs) availability, microbial diversity, and carbon dynamics in the soil?

If not, non‐assimilated polymers, which are probably subject to transformation and conversion into simpler substances, can be assimilated by other groups of microbes?

ii Which types of soil microbes predominate in contaminated soils and what characteristics do they have? Are they ecologically classified as copiotrophic or oligotrophic taxa?
iii How does the biodegradation of pollutants affect the quantity and quality of SOC? Can it change the microbial carbon use efficiency (CUE), defined as a proportion of microbial biomass carbon to SOC?

Consequently, the present review article aims to address critical knowledge gaps regarding the biodegradation of MPs by soil microorganisms, with a particular focus on the interactions between soil carbon status and microbial dynamics in contaminated environments. Unlike previous descriptive studies, this review seeks to explore complex questions regarding microbial preferences for MP‐derived polymers under varying organic carbon availability, thereby enriching our understanding of microbial ecology in the context of MP pollution. Additionally, the investigation of predominant microbial taxa, specifically copiotrophic versus oligotrophic, provides valuable insights into community structure and function that have been underappreciated in the existing literature. This exploration aligns with “Goal 15: Life on Land” of the United Nations Sustainable Development Goals (SDGs), which emphasizes the protection, restoration, and sustainable use of terrestrial ecosystems, as well as efforts to halt and reverse land degradation and biodiversity loss. Specifically, understanding the dynamics of MPs and their impacts on soil health is crucial for restoring ecosystem functions, which are essential components of sustainable land management practices.

To address important knowledge gaps regarding the ecological impacts of MP biodegradation in soil ecosystems, a systematic literature review was conducted using a predefined method for selecting, comparing, and synthesising relevant studies. This thorough search covered several reputable databases and used specific keywords related to MPs, soil microorganisms, biodegradation, and carbon cycling. Clear inclusion criteria were established, focusing on peer‐reviewed articles published between 2015 and 2025, resulting in a strong dataset of empirical studies examining the effects of MPs on soil microbial communities. By systematically comparing findings, common themes and differences in microbial responses were identified, especially in relation to soil organic carbon availability and its effect on microbial preferences for MP‐derived substrates. This careful approach provides a solid basis for understanding the complex interactions between MPs and soil microorganisms.

## 2 Biodegradation Mechanisms of MPS in Soil Environments

The existing literature on strategies for removing MPs has revealed significant debate about the relative importance of biodegradation of MPs, highlighting the need for further exploration. Much of the current research primarily describes natural events or investigates the biodegradation potential of soil microorganisms by examining mechanisms such as colonisation, deterioration, assimilation, digestive fragmentation, transformation, and mineralization under laboratory conditions (Zhang et al. 2021; Li, Yang, et al. 2023). Furthermore, the applicability of many studies on the biodegradation capabilities of various microorganisms in the soil, including bacteria, fungi, and algae, in different environments polluted by MPs poses a challenge. The large surface area of MPs creates favourable ecological niches for microorganisms that use them as carbon sources. In particular, prominent bacterial phyla such as Firmicutes, Actinobacteria, Proteobacteria, and Bacteroidetes, along with the fungal phylum Ascomycota, thrive in these conditions in soil ecosystems (Muroi et al. 2016; Li, Yang, et al. 2023).

The enzyme and biological degradation mechanisms used by microorganisms are categorised into various strategies, one of which is biodeterioration through the disruption of the MP polymer both inside and outside the MP particles in response to certain secreted enzymes, such as urease, lipases, and protease (Thakur et al. 2023). Biofragmentation is another strategy employed by microorganisms, in which specific enzymatic reactions hydrolyze the polymeric structure of MPs while catalysing oxidation processes resulting in the production of free radicals, the formation of carbonyl and hydroxyl functional groups, and consequently biological cleaving of the MP polymer (Ali et al. 2021). Furthermore, microorganisms can also mediate active assimilation or mineralization mechanisms, in which they can use MP monomers as a carbon source for energy supply (Kjeldsen et al. 2018). However, unassimilated polymers are subjected to a transformation process, through which the enzyme‐catalysed conversion process can transform chemical compounds into simpler substances to be assimilated by similar or other microbes (Thakur et al. 2023).

In more detail, the MPs biodegradation approach comprises several biochemical processes, in which the synthesis and the secretion of various types of intracellular and extracellular enzymes (e.g., esterase, urease, lipase, peroxidases, protease, carboxylesterases, glycoside hydrolases, keratinase and laccase) by microorganisms play a crucial role in biodegradation (Yuan et al. 2020). Such enzymatic reactions are particularly pronounced through bonding to the backbone of long‐chain MPs and splitting such long‐chain into monomer fragments, since high‐molecular‐weight MPs are not easily utilised or absorbed by microorganisms (Thakur et al. 2023). There is synergistic cooperation between intracellular enzymes synthesised by microbes and secreted extracellular enzymes, in which the former can induce the breakdown of stored endogenous carbon and the latter induces changes in the exogenous carbon pool. This cooperation results in the breakdown of MPs into more undersized molecules and fragments such as oligomers, dimers, and monomers (Iram et al. 2019). These small molecules can be metabolised into carbon dioxide and water by intracellular enzymes during hydrolytic and oxidative degradation, which can be considered processes that provide energy to microorganisms (Zhang et al. 2021). Therefore, it can be assumed that the activity of a single enzyme does not lead to complete MP biodegradation that requires the synergistic action of different enzymes (Li, Yang, et al. 2023). Such synergistic cooperation exists even between microorganisms, which can improve the efficiency of MP biodegradation compared to the effect of a single microorganism, as a consortium of microorganisms can obliterate the toxicity of metabolites secreted by a single strain during degradation (Lin et al. 2022; Li, Yang, et al. 2023).

**FIGURE 1:**
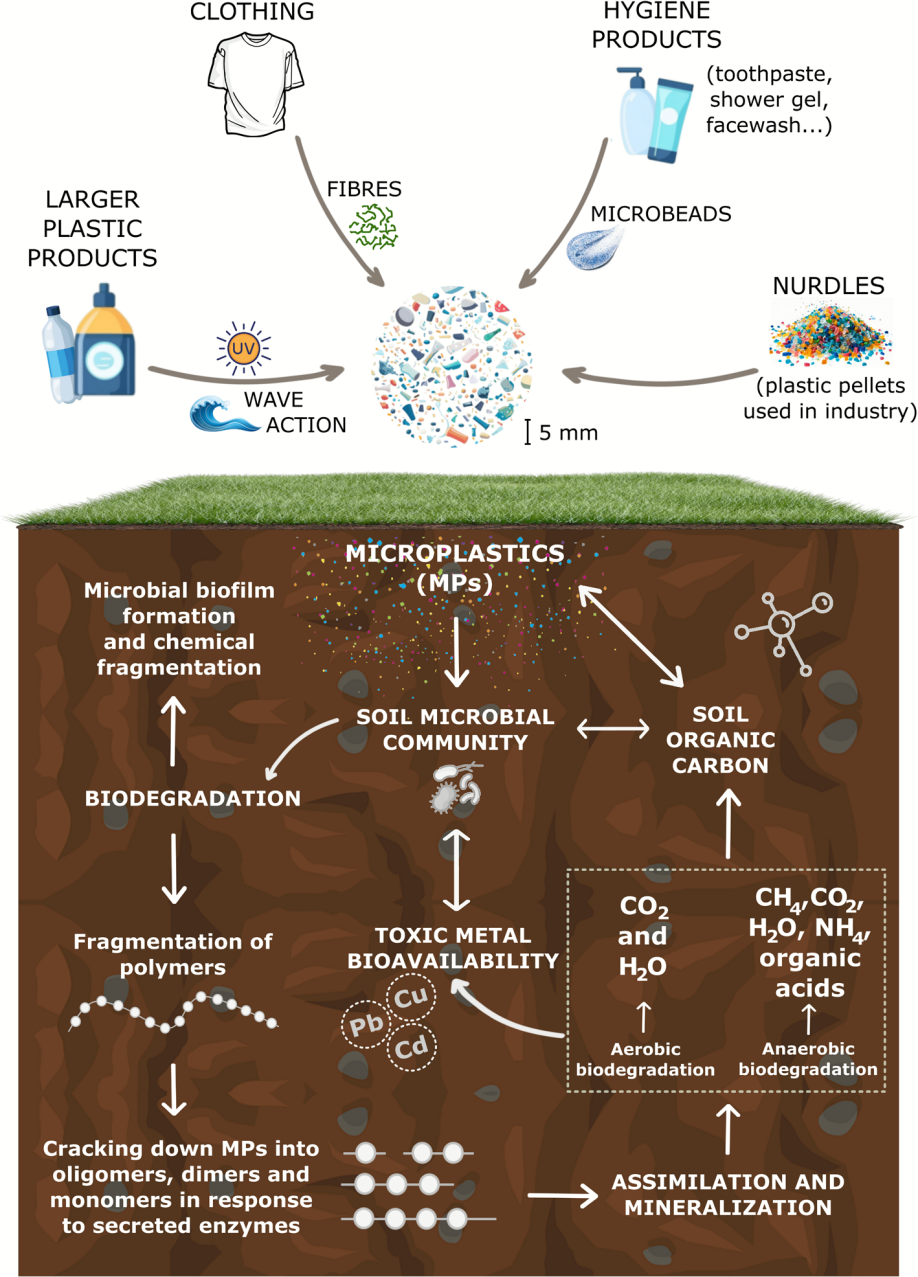
The pathways of MPs biodegradation by soil microorganisms, highlighting the stages of biofilm formation, fragmentation, and the subsequent aerobic and anaerobic degradation processes leading to assimilation and mineralization.

In addition, MPs can be assimilated and converted into methane, carbon dioxide, organic acids, water, and ammonium under anaerobic conditions to promote microbial growth, which not only is energetically unfavourable but can also require a considerably longer period to complete the mineralization process compared to aerobic conditions (Gu 2003). The ability of some microorganisms in MP biodegradation and their strategies used are presented in Table 2. The schematic of these pathways of MP biodegradation by soil microorganisms, including the stages of biofilm formation, fragmentation, and subsequent aerobic and anaerobic degradation processes that ultimately lead to assimilation and mineralization, is presented in Figure 1.

**TABLE 2:** MPs biodegradation by microorganisms and their mechanisms of action.

Microorganism		MPs	Finding	Mechanism of action	References
Bacteria					
Phylum	Species				
Firmicutes, *Proteobacteria* and Actinobacteria	*Bacillus cereus* , *Alcaligenes faecalis* , *Bacillus sonorensis* , *Staphylococcus epidermidis, Burkholderia vietnamensis*, *Rhodococcus ruber* , *Bacillus flexus* , *Sporosarcina globispora* , and *Bacillus gottheilii*	PET and PS	An 18% weight loss in MPs confirms their biodegradation by bacteria. Si, S, and Fe content was increased in the soil after biodegradation while decreasing C, Na, Mg, Al, Cl, and K.	Decrement in pH through the accumulations of organic acids during biodegradation in response to microbial activity and enzyme production, which in turn resulted in the oxidative or hydrolytic breakup of the ester or amide bonds.	Auta et al. (2022)
*Proteobacteria*	*Klebsiella* sp.	PVC	An acceptable bacterial survival rate on PVC biofilm, as the sole carbon source, was observed after 90 days of incubation.	The biodegradation mechanism was actuated by activating some related enzyme systems (e.g., esterase, lipase, catalase‐peroxidase, laccase, monooxygenase, and carboxyl esterase) and high expression levels of some genes encoding the proteins involved in the starvation response, oxidative stress, and nutrient transporter/assimilator cycles.	Zhang, Peng, et al. (2022)
Proteobacteria	*Ideonella sakaiensis*	PET	The high degradation efficiency was recorded in converting PET into ethylene glycol and terephthalic acid, as environmentally benign monomers.	Proteins synthesised and secreted by bacteria, named PET degradation enzymes (PETase), accompanied by a preliminary reaction mediator, called mono(2‐hydroxyethyl) terephthalic acid, were activated to biodegrade the PET.	Yoshida et al. (2016)
Firmicutes, proteobacteria and actinobacteria	*Bacillus cereus, Cytobacillus gottheilii*, *Enterococcus faecium, Listeria innocua, Exiguobacterium sibiricum, Cupriavidus necator, Burkholderia cepacia*, *Rhodococcus ruber*, and so forth	PS	Great potential in diverse earth environments and microbiomes was found for PS biodegradation by bacteria.	PS biodegradation through breaking down C–C bonds using three secreted enzymes by bacteria including alkane hydroxylases, monooxygenases, and cytochrome P450s.	Hou and Majumder (2021)
Proteobacteria	*Ideonella sakaiensis*	PET	Deconstructing PET into its constituent monomers in response to bacterial activity.	PET biodegradation in response to two‐enzyme systems synthesised by bacteria, including PETase and MHETase, which are involved in PET depolymerizing and 4‐methyl‐5‐hydroxyethyl thiazole (MHET) hydrolyzing, respectively.	Knott et al. (2020)
Firmicutes	*Bacillus* sp. and *Paenibacillus* sp.	PE	The dry weight and mean diameter of PE particles were reduced by 15% and 23% after 60 days of the biodegradation period, respectively.	PE particles were biodegraded by enzymatic chain scission, suggesting they could be biologically used as the only carbon source by bacteria.	Park and Kim (2019)
Firmicutes	*Exiguobacterium* sp.	PS	60 days of exposing PS particles to biodegradation resulted in their surface defects, such as wrinkles, holes, and notches.	Biodegradation was activated by bacteria by employing an oxygenase enzyme acting on the aromatic ring of PS.	Parthasarathy et al. (2022)
Firmicutes and Actinobacteria	*Bacillus* sp. and *Rhodococcus* sp.	PP	PP particles mass was reduced by 6% and 4% in response to *Rhodococcus* sp. and *Bacillus* sp., respectively, confirming the utilisation of PP for growth.	PP particles was degraded by bacteria since it was used for bacterial colonisation as a carbon source.	Auta et al. (2018)
*Proteobacteria*	*Pseudomonas* sp.	PS	Morphological and chemical modifications in PS were induced by bacteria during 60 days of biodegradation.	Secretion of serine hydrolase enzyme, a type of PETase, was suggested as the main designated enzyme for PS biodegradation, affecting the oxidation pathway through the formation of carbonyl groups.	Kim et al. (2020)
*Proteobacteria*	*Achromobacter denitrificans*	PVC	Shifting in the chemical and surface properties of PVC were recorded in response to bacteria.	An extracellular enzymes was found as a responsible for PVC biodegradation	Rad et al. (2022)
*Proteobacteria*	*Vibrio* spp., *Alteromonas* sp. and *Cobetia* sp.	PVC	Morphological and chemical alterations in PVC surface were observed during the biodegradation, in which the changes in the crystallinity, wettability, and tensile strength were more pronounced.	The ability of bacteria in PVC biodegradation was proposed using the utilisation of carbon from PVC for colonisation and the increase in dechlorination by enzyme synthesising.	Khandare et al. (2021)
Fungus and algae					
Ascomycota	*Aspergillus* sp.	PU	A 20% of degradation efficiency in PU was recorded in response to fungus.	Esterase enzyme activity was observed for the hydrolysis of PU, confirmed by the formation of calcium complex during biodegradation.	Osman et al. (2017)
Ascomycota	*Aspergillus niger*, and *Aspergillus fumigatus*	PP	PP biodegradation was confirmed by declining the weight of PP particles up to 71 and 53% in response to *A. niger* and *A. fumigatus* , respectively.	Not reported.	Williams and Osahon (2021)
Ascomycota	*Zalerion maritimum*	PE	The fungus induces PE biodegradation leading to a decrement in the mass and size of PE while requiring minimum nutrients for growth.	The biodegradation ability of the fungus may have something to do with the lower content of proteins and lipids and higher carbohydrate content of *Z. maritimum* when exposed to PE particles compared to control fungi.	Paco et al. (2017)
Ascomycota	*Fusarium oxysporum*, *Fusarium falciforme* and *Purpureocillium lilacinum*	PE	Morphological changes in PE surface were found in response to fungi, such as bumps, pits and furrows, swellings, and partial exfoliations.	Activating the oxidation mechanisms by fungi and targeting the methyl terminal groups of PE.	Spina et al. (2021)
Chlorophyta	*Scenedesmus abundans*	PS, PMMAand PLA	The removal efficiency of PMMA, PLA, and PS by microalgae was recorded at about 98%, 87%, and 84%, respectively.	This result was explained by the hetero‐aggregation between *S. abundans* and microplastics, as well as the increased adsorption of microplastics onto the container wall	Cheng and Wang (2022)

Abbreviations: PE, polyethylene; PET, polyethylene terephthalate; PLA, polylactide; PMMA, poly(methyl methacrylate); PP, polypropylene; PS, polystyrene; PU, polyurethane; PVC, polyvinyl chloride.

## 3 Microbial Preferences for Carbon Sources

MPs, primarily composed of carbon, can serve as soil carbon storage and potentially influence the soil organic matter composition and its turnover of soil organic matter; however, their rates of entry and effects on ecosystems remain unclear (Rillig and Lehmann 2020). Recent studies suggest that MPs play a crucial role in the accumulation and stability of soil carbon storage through interactions with various forms of soil carbon, including both particulate and mineral‐associated organic matter (Chen et al. 2024). Furthermore, MPs alter the chemical diversity of dissolved organic matter in soils by increasing its aromaticity, molecular weight, and degree of humification (Chen et al. 2023). These transformations are intricately linked to the interactions among MPs, organic materials, and microbial biodegradation processes (Chen et al. 2024). Consequently, MPs create unique ecological niches that significantly influence microbial community dynamics and pathways of necromass formation, thereby impacting soil carbon cycle dynamics and contributing to shifts within ecosystems (Camenzind et al. 2023). For instance, Rillig et al. (2021) proposed three hypotheses concerning the interaction between MPs and the decomposition of SOC: (i) electron shuttling, where MPs may enhance SOC breakdown by attracting microorganisms that utilise them as electron donors; (ii) microbial frustration, where the presence of MPs potentially increases the diversity of resistant carbon compounds, thereby inhibiting microbial decomposition; and (iii) priming, in which biodegradable MPs promote SOC decomposition as a labile carbon source (positive priming), while nondegradable MPs might hinder SOC mineralization by sequestering dissolved organic carbon (negative priming). This last hypothesis illustrates the intricate relationships between microbial preferences for carbon sources and the ecological ramifications of MP contamination, demonstrating that while conventional MPs could limit the contribution of microbial necromass to the stable soil carbon pool, biodegradable MPs may enhance it (Rillig et al. 2021; Chen et al. 2024).

However, it is essential to approach the understanding of microbial preferences for carbon sources with caution, especially regarding monomers derived from MPs as potential energy substrates. Microbial community composition, environmental parameters, physicochemical attributes of carbon sources, and the biochemical characteristics of available substrates play critical roles in shaping microbial utilisation behaviours (Baldrian 2017; Yaghoubi‐Khanghahi et al. 2020; Martínez‐Toledo et al. 2021; Addesso et al. 2023). The availability of monomeric carbon sources from MPs may not easily predict microbial responses, suggesting an obligation to consider all these ecological dynamics to expect changes in microbial activity and community structure. Thus, while MPs consist of approximately 90% carbon (Rillig and Lehmann 2020), they often lack the functional properties of natural soil organic matter, primarily due to their slow decomposition rates, which limits their effectiveness as carbon substrates for soil microorganisms (Rillig et al. 2021).

Importantly, the non‐assimilated polymers resulting from MP degradation may not be fully utilised by the original microbial communities responsible for their breakdown; rather, they might be transformed by other soil microorganisms into simpler, more readily usable forms, integrating into the microbial loop and highlighting the complexity of microbial interactions within the soil food web. Therefore, while the degradation of MPs presents ecological challenges, it may also lead to the generation of new carbon sources accessible to diverse microbial communities, ultimately influencing organic matter turnover (Ju et al. 2023). A key consideration regarding the role of microorganisms in the soil carbon cycle is the intense competition between bacteria and fungi for readily accessible carbon and energy sources. Both groups have developed various competitive and symbiotic strategies for decomposing resistant organic materials (de Boer et al. 2005). When SOC is abundant, microorganisms tend to preferentially utilise these organic compounds due to their higher energetic yield and the diversity of available nutrients (Wang and Kuzyakov 2024). Nevertheless, certain microbial taxa, including specific Gram‐negative bacteria, have adapted to degrade MPs and may preferentially target the monomers released from MPs, even in the presence of SOC (Wang and Kuzyakov 2024). In this context, the next section elaborates on the concept that certain microorganisms possess the ability to degrade MPs, indicating a niche adaptation that allows them to utilise less conventional substrates as alternative sources of carbon.

In sum, this discussion lays the foundation for understanding the multifaceted interactions between MPs and soil microbes, establishing a connection to the upcoming sections on how these plastics impact microbial dynamics in light of environmental changes, the role of co‐metabolism, and the shifts in ecosystem dynamics associated with MP degradation.

## 4 MPs and Soil Microbes: Copiotrophs Thriving Amidst Environmental Change

As MPs accumulate as pollutants in soil, they influence the intricate balance of microbial communities that are crucial in nutrient cycling and soil fertility. The MP‐degrading capability of some microorganisms suggests a niche adaptation, enabling these organisms to exploit less common substrates as an alternative carbon source. In this context, r‐strategist (copiotrophic) organisms, often termed “opportunistic,” exhibit rapid growth rates and high reproductive capabilities that allow them to quickly absorb readily available substrates, positioning them advantageously in MP‐contaminated environments. Their ability to exploit these conditions facilitates the initial biodegradation of synthetic polymers. Conversely, slow‐growing K‐strategist organisms, typically represented by fungi and Gram‐positive bacteria, grow more slowly and excel at utilizing complex organic compounds (Fierer et al. 2003; Wang and Kuzyakov 2024). This distinction is evident in the rhizosphere, where plant roots release low molecular weight substrates, which typically has a significantly higher density of bacteria, particularly those Gram‐negative, compared to fungi (Jones 1998). Moreover, it has been proposed that simple carbon sources released by plant roots such as amino acids and glucose can boost the abundance of Gram‐negative bacteria in the soil as compared to other microorganisms (Chen et al. 2022).

Recent studies have reported that soils contaminated with MPs exhibit elevated abundances of Proteobacteria, Actinobacteria, and Bacteroidetes (Huang et al. 2019; Zhang, Li, et al. 2023). These bacterial groups exhibited a positive correlation with the carbon content in the soil, leading to their classification as copiotrophic bacteria (Fierer et al. 2003). In a study by Liu et al. (2023), various global change factors (e.g., microplastics and heavy metal pollution) were assessed, indicating that these factors can diminish microbial diversity and subsequently decrease soil oligotroph/copiotroph ratio. Zhang, Li, et al. (2023) reported that MPs affected microbial diversity and function in contaminated soil by increasing microbial nitrogen requirements, reducing organic carbon mineralization, and driving changes through soil nutrient availability and oxidative stress. Moreover, it has been reported that MPs exert a more pronounced influence on fungal communities compared to bacterial communities, as evidenced by a significant decline in arbuscular mycorrhizal fungi (AMF) abundance and an increase in ectomycorrhizal fungi following MP addition. Additionally, while MPs can enhance enzyme activities and elevate the abundance of certain functional genes in soil bacteria, these changes do not fully account for the observed shifts in microbial community dynamics, being fungal diversity particularly sensitive to the impacts of MPs (Fan et al. 2022). Nevertheless, it is important to recognise that the influence of MP pollution on microbial communities in soil is complex and multifaceted. While certain microbial phyla are commonly categorised as copiotrophic or oligotrophic, it should be noted that not all members within these phyla demonstrate uniform responses to environmental changes. The differential effects of microplastic contamination can lead to nuanced interactions among microbial taxa, complicating our understanding of community dynamics. Consequently, a comprehensive evaluation of microbial response to MP pollution must consider these intricate relationships and the variability in metabolic strategies among individual microbial species.

## 5 The Role of Co‐Metabolism in Polymer Degradation by Soil Microorganisms

The phenomenon of co‐metabolism plays a significant role in the bioremediation of persistent pollutants, particularly in soil environments where microbial activity is vital for the degradation of recalcitrant compounds. Co‐metabolism is a microbial process wherein certain microorganisms can utilise specific substrates necessary for their growth, known as primary metabolic substrates, while concurrently and non‐specifically altering additional compounds that are not essential for their growth, referred to as co‐metabolic substrates. In this process, the co‐metabolised substances are typically not relied upon as sources of energy or carbon for the microorganisms; rather, they are transformed through the action of the microorganisms' enzymatic systems (Zhao et al. 2024). This definition outlines the process of co‐oxidation and/or co‐reduction of a substrate facilitated by whole cells that use non‐specific enzymes, even though those cells do not obtain energy or nutrients from the transformed substrates for their growth (Hazen 2010). This process can be relevant in the degradation of polymers, which are often resistant to microbial attack because of their complex chemical structures; however, the effects of co‐metabolism in this context remain understudied. In the presence of more easily degradable organic substrates, soil microorganisms can perform co‐metabolism, facilitating the degradation of polymers that they otherwise could not use effectively. For example, when a microorganism utilises readily available carbon sources, such as sugars or organic acids, it may also promote the enzymatic processes necessary to break down synthetic polymers. This process is exemplified by various bacterial species that possess the enzymatic machinery to initiate polymer degradation, relying on co‐metabolic pathways to do so. It has been reported that co‐metabolism plays a significant role in the biodegradation of MPs by stimulating the growth of r‐strategist microorganisms, particularly Gram‐negative bacteria (Wu et al. 2025). Moreover, the initial degradation of MP particles promotes the turnover of SOC through microbial‐mediated processes, by releasing available carbon substrates that serve as nutrients, thereby further enhancing the growth of fast‐growing microbial groups (Nelson et al. 2022; Wu et al. 2025). This dynamic interaction results in a positive priming effect driven by microbial co‐metabolism, wherein the activity of copiotrophic (r‐strategist) microorganisms is elevated, leading to accelerated biodegradation of the MPs and improved soil health (Rillig et al. 2021). Research has demonstrated that adequate initial soil nutrients, such as SOC and dissolved organic carbon, combined with optimal soil carbon‐to‐nitrogen (C/N) ratios, can fulfil the microbial stoichiometric nutrient requirements and enhance the co‐metabolism of both exogenous and native organic carbon. This process, in turn, contributes to the stabilisation of the deep soil carbon pool (Gaudel et al. 2022). Overall, understanding the role of co‐metabolism in soil microbial communities can provide valuable insights into enhancing polymer degradation strategies, highlighting the importance of nutrient dynamics and microbial interactions in the bioremediation of synthetic pollutants.

## 6 The Hidden Ecological Risks: Challenges of Incomplete MPs Biodegradation

On the other hand, because complete biodegradation of MPs cannot be achieved in realistic environments, even for biodegradable plastics, the risk of more severe MP contamination in soil ecosystems during biodegradation cannot be overlooked (Qin et al. 2021). It is important to remember that the ecological effects of persistent MPs may differ from those of biodegradable MPs. While the latter may have some advantages regarding breakdown, their incomplete biodegradation introduces unique risks that may not be apparent when evaluating the effects of persistent MPs. Despite this, little progress has been made in revealing the fate of MPs in terms of the accumulation of environmental pollutants (e.g., potentially toxic metals and persistent organic pollutants) after bio‐decomposition. Shifts in the mobility of toxic metals in soil can be expected because of a considerable correlation among the presence of MPs, heavy metals availability, and microbial diversity and functions in soil (Feng et al. 2022), as a change in one factor can cause a modification in others. This theoretical concept has led to investigations analysing and reporting the resulting potential ecotoxicological threats of incomplete biodegradation of MPs, which can result in increased accumulation and availability of toxic chemical compounds and heavy metals (Qin et al. 2021; Chah et al. 2022; Shi et al. 2023). For instance, a higher absorption/release characteristics of heavy metals (e.g., Pb, As, and Cr) upon degradation of biodegradable MPs, polybutylene adipate‐co‐terephthalate, was reported (Li et al. 2020). Such an ecological risk is more pronounced during the incomplete biodegradation of bioplastics and biodegradable MPs compared to non‐degradable MPs, which can lead to a higher and more uneven surface area and therefore boosts their capability to interact and adsorb greater quantities of contaminants (Qin et al. 2021; Chah et al. 2022). The debates about plastic biodegradation have acquired prominence regarding the proper time for a complete biodegradation process to avoid the contamination risk and how this can be estimated (Kyrikou and Briassoulis 2007).

It is essential to consider such challenges in the efficiency of biodegradation by soil microorganisms since these biological pathways depend on several factors, including the nature and availability of the microorganisms, the relative abundances of the beneficial biodegrading microorganisms in the soil, and their potential for colonisation, as well as environmental conditions (soil pH, moisture, oxygen and temperature) (Yogalakshmi and Singh 2020; Golmohammadi et al. 2023; Thakur et al. 2023). Moreover, most MP polymers are biologically resistant to degradation or are just partially biodegradable because of their high molecular weight, hydrophobic characteristics, and lack of functional groups compatible with microbial enzymatic reactions (Yogalakshmi and Singh 2020). Given all that has been mentioned so far about the risk of accumulation of pollutants in response to incomplete biodegradation, several questions remain unanswered at present. Therefore, there is significant potential for further research in determining the fate of harmful byproducts (e.g., toxic metals) upon the biodegradation by microorganisms and answering the questions that have been raised about the safety of prolonged use of MPs biodegradation strategy.

Incorporating the insights from the previous discussion, it is essential to recognize that incomplete MP degradation can significantly influence microbial adaptation and community dynamics in affected ecosystems through several ecological perspectives. Firstly, the presence of MPs in the environment provides new surfaces and substrates for microbial colonisation, leading to changes in community structure (Li, Yang, et al. 2023). As discussed in the previous sections, microorganisms may adapt to utilise MPs as alternative energy or nutrient sources, which can select specific microbial taxa capable of utilising these compounds, resulting in shifts in microbial diversity. Additionally, the accumulation of heteroatom‐containing compounds during the incomplete degradation process can create selective pressures that favour microorganisms with specialised metabolic pathways (Gundlapalli et al. 2024). These adaptive responses can enhance microbial resilience and promote the development of distinct microbial communities that are more proficient in degrading or assimilating MPs and their degradation products. Furthermore, biofilm formation on the surfaces of MPs can influence microbial interactions, leading to cooperative behaviours or competitive dynamics among different species (Jia et al. 2024). Such interactions can facilitate nutrient cycling or enhance the biodegradation process, ultimately impacting ecosystem functions.

## 7 Shifts in Ecosystem Dynamics due to MPS Degradation

MP biodegradation in soils can lead to significant alterations in both physical and chemical properties (Santini, Acconcia, et al. 2022; Chen et al. 2024), critically impacting the soil ecosystem, including its fauna and microbiome (Santini, Zizolfi, et al. 2024; Chen et al. 2024). Degraded MPs can affect soil structure by creating aggregates that alter pore size distribution; this alteration influences water retention capabilities and aeration, which are essential for root health and microbial respiration (Sajjad et al. 2022). Poor aeration may result in anaerobic conditions, consequently affecting the metabolic processes of soil organisms and potentially resulting in shifts in microbial communities towards those that are less beneficial for plant growth (Qian et al. 2022). Moreover, the chemical leaching of additives and degradation byproducts from MPs into the soil can change nutrient availability, pH levels, and overall soil chemistry, further influencing the function and diversity of microbial communities (Sajjad et al. 2022; Santini, Probst, et al. 2024). This chemical disruption can impact the symbiotic relationships between soil microbes and plants, leading to reduced nutrient uptake and impaired soil health. Consequently, the cascading effects of MP transformation on soil physical and chemical properties underline the need to understand these interactions to develop effective strategies for soil remediation and conservation in the face of increasing plastic pollution.

As microorganisms actively decompose plastics, the complex interactions between soil nutrients and these newly released compounds can significantly modify the availability of key nutrients that are critical for microbial growth and activity (Yang and Chandran 2021). These changes may create conditions that favour specialised microbes capable of degrading MP, potentially resulting in their dominance within a niche, which could challenge the presence of more generalist microorganisms (Rillig et al. 2021). Such competitive displacement could result in a decline in microbial diversity (Yan et al. 2021), which is concerned with a lower diversity that can reduce ecosystem resilience and functionality, and impair the ecosystem's ability to respond to environmental changes or disturbances. Moreover, a reduction in microbial diversity can negatively impact nutrient cycling processes and soil structure, further aggravating the effects of MP pollution and undermining the overall integrity of the ecosystem (Jain et al. 2023).

If microorganisms prioritise the utilisation of monomers derived from MPs over SOC, this shift could lead to profound modifications in soil ecosystem dynamics. The degradation of MPs by various microbial communities can release important micronutrients into the environment, but it also carries risks by potentially introducing toxic compounds and PTEs. These changes can alter soil chemistry and negatively affect overall soil health (Feng et al. 2022). Moreover, MPs with large surface areas can absorb essential nutrients in the soil (Jia et al. 2023), thereby affecting soil fauna, plant growth, and soil microbiome structure. Additionally, this absorption can interfere with the natural processes of nutrient cycling and energy transfer among microbial communities, leading to cascading effects on the soil ecosystem (Jain et al. 2023).

Although extensive studies have examined how plastic affects microbial communities in their natural environments, it is also important to recognise that environmental pollutants significantly influence the movement and distribution of microbes between different habitats (Lear et al. 2021). Buoyant plastics like polyethylene, polypropylene, and polystyrene can be carried over great distances by wind and ocean currents, while heavier plastics, such as polyethylene terephthalate and polylactic acid, may transport microbes attached to their surfaces across various ecosystems (Lacerda et al. 2019; Audrézet et al. 2020). This displacement can include invasive and non‐native microbial species such as potential pathogens, antibiotic‐resistant microbes, and harmful microalgae that affect the host microbial community (Moore et al. 2020; Lear et al. 2021).

Moreover, genomic investigations into microbial communities interacting with MPs revealed insights into how these organisms adapt to their novel environment, including the acquisition of specific genes related to the degradation of plastic compounds. Microorganisms alter their gene expression in response to exposure to environmental pollutants and stressors, such as MPs, either directly or indirectly (Tang et al. 2024). Recent studies have highlighted significant differences in gene expression profiles between microbial communities inhabiting MPs and those in the surrounding environment, indicating that certain subcategories of genes showed varying signal intensities, suggesting complex interactions that affect microbial metabolism (Rahman et al. 2021; Agathokleous et al. 2021; Chen et al. 2025). For instance, genes associated with carbon fixation were found to be highly expressed in MP‐associated communities, while others displayed elevated expression in the surrounding environment, further complicating our understanding of microbial adaptations (Rahman et al. 2021; Li, Jia, et al. 2023). Moreover, it has been reported that long‐term MP contamination in cropland soils influences the distribution of heavy metal resistance genes in soil microorganisms, forming a modular distribution of these genes that are associated with heavy metals, such as Cu and As, and affecting soil ecological security (Wu et al. 2023). Several other gene categories, including those related to carbon cycling, metal homeostasis, nitrogen fixation, phosphorus and sulfur cycling, pollutants remediation, and secondary metabolite production, were also reported as being highly expressed in response to MP contamination (Rahman et al. 2021). MPs can also activate genes associated with oxidative stress and the inflammatory response, resulting in increased ROS production, while also altering gene expression in other biological processes (Tang et al. 2024). Research has shown that MPs can affect chromosomes, DNA, and gene expression, with connections to inflammation and oxidative stress (Tang et al. 2024). It has been proved that MPs can cause clastogenesis in microbes, resulting in chromosome damage, and leading to failures in chromosome segregation without causing direct damage (Tang et al. 2024). Additionally, MPs may epigenetically influence chromosomes by disrupting the mechanisms that govern their proper segregation and altering their structures or associated proteins, all causing no direct damage (Poma et al. 2023). Epigenetics explores heritable changes that do not involve DNA mutations, explaining how identical genomic instructions can lead to diverse cell types within an organism, and it plays a crucial role in gene expression regulation, developmental processes, and the adaptability of organisms to environmental stimuli (Holliday 2006).

Nonetheless, it is important to recognize the differences between persistent and biodegradable MPs, since their microbial degradation processes and ecological impacts differ, even though the distinctions between these categories are not always clearly defined. In this regard, it has been reported that MPs in soil impact gene abundance related to carbohydrate and energy metabolism, with biodegradable MPs exerting a stronger effect than traditional types (Sun, Duan, Cao, Ding, et al. 2022; Chen et al. 2025). Moreover, biodegradable MPs alter the microbial gene pool's composition and diversity, enhancing the secretion system and transport protein genes (Rüthi et al. 2023). However, they may also promote overexpression of genes linked to the carbon‐nitrogen cycle and antibiotic resistance, as MPs can serve as habitats for pathogens (Li, Jia, et al. 2023). Understanding these molecular adaptations and variations in gene expression is essential for developing strategies to mitigate the ecological impact of MPs, particularly as we navigate the complexities posed by their diverse forms and behaviors in various environmental contexts.

## 8 Linking MPS Biodegradation to Microbial Carbon Use Efficiency

It has been suggested that the biodegradation of MPs can influence microbial carbon use efficiency (CUE) (Tang et al. 2023). CUE refers to the ability of microorganisms to convert organic carbon into microbial biomass relative to the amount of carbon they respire as CO_2_ (Yaghoubi‐Khanghahi et al. 2020). The efficiency of this process is intricately linked to the quality and complexity of the substrates (Yaghoubi‐Khanghahi et al. 2019). It has been reported that readily biodegradable pollutants, such as simple carbohydrates or low‐molecular‐weight organic acids, often result in elevated CUE (Adingo et al. 2021). Microorganisms can utilise these compounds with greater ease, leading to more efficient biomass production. Conversely, microbial communities are often compelled to expend considerable energy on degradation processes without a proportional increase in biomass when faced with more recalcitrant pollutants, such as long‐chain hydrocarbons or complex aromatic compounds (Alaidaroos 2023; Picariello et al. 2022). As discussed above, MP particles present in the soil can alter the structure and the ecological functioning of microbial communities, impacting essential processes like organic matter decomposition and nutrient cycling. Such alterations may influence microbes' CUE and introduce metabolic limitations, leading to reduced carbon utilisation by soil microbes, which in turn may decrease soil nutrients and hinder the overall functionality of the ecosystem (Tang et al. 2023). This inefficient metabolic pathway can lead to a clear decrease in CUE, as microorganisms struggle to extract energy while maintaining growth (Regueira et al. 2021).

Furthermore, the bioavailability of nutrients plays a key role in shaping microbial activity and CUE (Mganga et al. 2022). As microorganisms must balance their need for cellular growth with the energy expended in degrading complex pollutants, limiting nutrients (e.g., nitrogen or phosphorus) can further exacerbate the inefficiencies in carbon utilisation (Manzoni et al. 2012). Therefore, as mentioned earlier, since conventional MPs may reduce the availability of nutrients in the soil (Sajjad et al. 2022), their negative impact on carbon utilisation efficiency is expected. The effects of other unfavourable environmental factors on CUE should also be considered, which can further reduce the CUE in the conditions of MP pollution. Accordingly, environmental conditions such as oxidative or reductive states also impact microbial metabolic pathways, altering microbial community compositions and their ecological functions (Bouranis and Tfaily 2024). The influence of other environmental factors, such as high temperature (Allison et al. 2010) and water stress (Hagerty et al. 2014) related to the current pressing climate change, can also reduce the carbon absorbed for microbial growth and consequently decline the CUE.

Nevertheless, discussing the effects of MPs on microbial CUE can be much more complex and generalising the results should be approached with caution. Studies have shown that biodegradation rates vary significantly among different types of MPs, subsequently influencing the efficiency of microbial communities in carbon utilisation (Song et al. 2025). In this regard, it has been proposed that different types and concentrations of MPs, especially those biodegradable, would variably affect microbial CUE and nutrient cycling, driven by differences in microbial community composition and functional dynamics (Song et al. 2025). Notably, the studies have elucidated correlations between biodegradation rates and alterations in microbial biomass and community composition, revealing their substantial effects on SOC dynamics (Rauscher et al. 2023). Consequently, quantitative assessments of CUE in relation to specific rates of MP degradation have provided valuable insights into how microbial metabolic processes are shaped by varying concentrations of MPs and their biodegradability. For instance, research has shown that the degradation of polyhydroxyalkanoates (PHA) can enhance SOC mineralization due to their readily biodegradable nature, promoting microbial activity and growth (Yean et al. 2017; Suzuki et al. 2021). In contrast, higher concentrations of polylactic acid (PLA) lead to increased CO_2_ emissions, primarily due to their slower degradation rates caused by higher molecular weight, which restrict the accessibility for microbial utilisation (Emadian et al. 2017; Mosomi et al. 2024). Polybutylene adipate terephthalate (PBAT) exhibits minimal effects on SOC dynamics, likely because their slow degradation similarly limits interactions with soil microbes (Song et al. 2025). On the other hand, biodegradable poly(3‐hydroxybutyrate‐co‐3‐hydroxyvalerate) (PHBV) is readily utilised by microbial communities as a carbon source, resulting in higher microbial growth rates and enhanced biomass activity in the microplastisphere (Zhou et al. 2021). Furthermore, the increased CO_2_ emissions associated with biodegradable MPs may arise not only from microbial degradation but also from a priming effect on soil organic matter (Zhang, Liu, et al. 2023). Understanding whether there is a link between CUE and the rate of biodegradation could reveal critical information about microbial preferences and efficiencies in utilising various carbon sources derived from MPs. It has been shown that microbial CUE increases with higher concentrations of biodegradable MPs (PHA) in soil (Song et al. 2025) as well as with conventional MPs in agroecosystems (Zang et al. 2020). This increase in CUE is attributed to two main factors: First, the addition of MP materials likely redirects carbon allocation within microbial cells towards maintenance needs. This shift is significant because CUE is influenced by both the quality and quantity of carbon substrates and the specific characteristics of the microbial community (Takriti et al. 2018; Silva‐Sánchez et al. 2019). Song et al. (2025) emphasised that soil carbon resources, enzyme activities, and microbial community composition are crucial determinants of CUE; their findings are further supported by the positive relationship between CUE and dissolved organic carbon levels, which suggests a shift towards utilising more readily available carbon sources. Secondly, the increase in microbial biomass and subsequent changes in community structure have also contributed to the rise in CUE (Silva‐Sánchez et al. 2019). The significant growth in microbial biomass in response to elevated MP levels, combined with a strong correlation between microbial β‐diversity and CUE, supports this hypothesis (Song et al. 2025). These findings indicate that MPs can profoundly influence soil carbon dynamics by altering the availability and quality of carbon substrates while simultaneously reshaping microbial community structures, with potentially different effects depending on the type of MP involved.

It can be concluded that the biodegradation process not only affects the dynamics of SOC quantity and quality but also reflects significant changes in microbial metabolic strategies and efficiencies. This highlights the intricate relationship between pollutant degradation, soil carbon dynamics, and microbial ecology in determining soil health and ecosystem functions. As microbial communities adapt to different pollutants and environmental pressures, they may exhibit shifts in their metabolic pathways, which can further influence the overall health of the soil ecosystem (Philippot et al. 2021). The present review states that the degradation of certain MP pollutants may stimulate the proliferation of specific microbial taxa that are adapted at metabolising those compounds, which may alter the soil microbial community structure and function. This dynamic interaction underscores the importance of considering both pollutant types and microbial responses in soil studies, as the microbial mechanisms underlying biodegradation can have widespread implications for ecosystem services such as carbon sequestration, nutrient cycling, and pollutant degradation. Continued research into microbial CUE in pollutant biodegradation will be essential for developing effective soil management practices and improving soil health in polluted environments.

## 9 Concluding Remarks

In conclusion, the present review aims to explain and elucidate the multifaceted role of soil microorganisms in the biodegradation of MPs, emphasising the interplay between soil carbon status and microbial community dynamics. The findings indicate that while MPs are predominantly composed of carbon, their structural properties limit their functional equivalence to that of natural soil organic matter, consequently affecting the degradation process and overall use as a carbon source for soil microorganisms. Some microbial taxa have developed varied strategies, ranging from competitive to symbiotic interactions, to thrive in environments contaminated with MPs. Compelling evidence suggests that even non‐assimilated polymers resulting from MP degradation might be transformed by other soil microorganisms into more readily utilisable forms, indicating significant microbial interactions within the soil food web that are crucial for carbon cycling. These findings underscore the importance of considering how microbial preferences for MP‐derived substrates are influenced by the availability of more accessible soil organic carbon. Moreover, the metrics of carbon use efficiency among these microbial communities highlight the variability in energy allocation towards biomass production versus respiration, showing the nuanced responses of soil microorganisms to different carbon sources, including those derived from MPs.

## 10 Limitations and Future Perspectives

This review acknowledges several key limitations, one of which is the predominance of laboratory‐based studies, which may not fully replicate the complexities and variations of real‐world soil ecosystems. Furthermore, the variability of microbial responses to MPs, influenced by environmental factors such as moisture, temperature, and soil type, remains unexplored. Future research should prioritise field studies to assess microbial interactions in diverse environmental contexts, using advanced molecular techniques to explore microbial community dynamics and functional capacities on a larger scale. Moreover, investigating the long‐term ecological effects of MP degradation, as well as the fate of resulting byproducts, will be important in developing effective bioremediation strategies and informing policies aimed at reducing plastic pollution in terrestrial ecosystems.

A more comprehensive understanding of how soil microorganisms interact with different carbon sources and the implications of these interactions on soil ecosystem health is essential for developing effective bioremediation strategies. By enhancing our knowledge in this area, we can better address the pressing environmental challenges posed by MP pollution and promote the sustainability of terrestrial ecosystems. Such insight will be crucial for informing practices that protect soil health and resilience in the face of increasing contaminant loads.

## Author Contributions

**Mohammad Yaghoubi Khanghahi:** conceptualization, investigation, writing – original draft, writing – review and editing. **Carmine Crecchio:** conceptualization, writing – original draft, writing – review and editing, investigation. **Adriano Sofo:** conceptualization, writing – original draft, writing – review and editing, funding acquisition, supervision. **Rosangela Addesso:** conceptualization, investigation, writing – original draft, writing – review and editing, funding acquisition.

## Funding

This study was carried out within the Agritech National Research Center and received funding from the European Union Next‐GenerationEU (PIANO NAZIONALE DI RIPRESA E RESILIENZA (PNRR)—MISSIONE 4 COMPONENTE 2, INVESTIMENTO 1.4—D.D. 1032 17/06/2022, CN00000022). This manuscript reflects only the authors' views and opinions, neither the European Union nor the European Commission can be considered responsible for them.

## Conflicts of Interest

The authors declare no conflicts of interest.

## Data Availability

Data sharing not applicable to this article as no datasets were generated or analysed during the current study.
